# Subjective Physical Performance and Its Determinants in Patients With Haemophilia

**DOI:** 10.1111/hae.70037

**Published:** 2025-03-28

**Authors:** Alexander Schmidt, Fabian Tomschi, Pia Möllers, Marius Brühl, Sylvia von Mackensen, Andreas C. Strauss, Heinrich Richter, Johannes Oldenburg, Thomas Hilberg

**Affiliations:** ^1^ Department of Sports Medicine University of Wuppertal Wuppertal Germany; ^2^ Department of Orthopaedics and Trauma Surgery University of Bonn Bonn Germany; ^3^ Department of Medical Psychology University Medical Centre Hamburg‐Eppendorf Germany; ^4^ Haemophilia Centre Muenster Muenster Germany; ^5^ Department of Experimental Haematology and Transfusion Medicine University Clinic Bonn Bonn Germany

**Keywords:** haemophilia, HEP‐Test‐Q, joint status, pain, rare disease, subjective physical performance

## Abstract

**Introduction:**

Physical functioning is compromised in patients with haemophilia (PwH). However, factors negatively influencing subjective physical performance (SPP) remain underexplored. Hence, this study aimed to compare the SPP of PwH with healthy controls (CON), to differentiate them based on disease‐specific, person‐related, and arthropathy‐related parameters, and to identify overarching determinants influencing SPP.

**Methods:**

SPP was assessed in 301 PwH and 263 CON via the HEP‐Test‐Q, which divides SPP into a total score and four distinct dimensions (e.g., mobility). Additionally, disease‐specific (i.e., type, severity, treatment regime, HIV, hepatitis), person‐related (i.e., age, BMI), and arthropathy‐related parameters (i.e., current pain intensity (NRS‐now) and average pain intensity across 4 weeks (NRS‐4w) and the HJHS) were examined, and associations with SPP were calculated.

**Results:**

All PwH and PwH subgroups demonstrated significantly greater impairment across all SPP dimensions compared to CON. Apart from the type of haemophilia (A vs. B, *p* = 0.894), significant differences in total SPP were observed among PwH subgroups for severity (severe vs. non‐severe, *p* = 0.012), treatment (prophylaxis vs. on demand, *p* = 0.002), HIV (no vs. yes), and hepatitis (no vs. yes, both *p* < 0.001). A multiple linear regression model further revealed significant predictive effects for HJHS (*p* < 0.001) and NRS‐4w (*p* < 0.001) on the total SPP score.

**Conclusion:**

PwH perceived their physical performance as significantly worse across all dimensions compared to CON. The decreased SPP in PwH can be attributed primarily to arthropathy‐related factors, that is, an impaired joint status and persistent pain. To oppose the decline in SPP, tailored sports‐therapeutic programs should be integrated into the multimodal treatment concept.

## 1 Introduction

Single or repeated exposure of intra‐articular structures to blood‐derived iron not only renders the joint more susceptible to recurrent bleeding events, but also evokes chronic pain and irreversible musculoskeletal alterations in patients with haemophilia (PwH) [1, 2, 3]. Conventionally, the ankle, knee, and elbow represent the most commonly affected joints [4]. Both chronic pain and pathophysiological joint changes can lead to maladaptation of the motor system [5]. Previous studies, mainly in younger patient populations, have demonstrated a reduction in upper and lower body dynamic strength [6, 7], single‐leg and bilateral isometric strength [8], aerobic and anaerobic capacity [9, 10], and deficits in static proprioception and coordination in PwH [8].

Considering that objective screening of all relevant motor skills is time‐consuming, requires qualified personnel, and may not be feasible given arthropathy‐related joint constraints, the Haemophilia and Exercise Project‐Test‐Questionnaire (HEP‐Test‐Q) was designed as a subjective measurement tool to assess physical performance in PwH [11]. The subjective perception of physical performance showed moderate to high correlations with the objective assessments (e.g., one‐leg‐stand), and the authors concluded complementary benefits of implementing both methods [12]. Analogous to objective measures, studies in which the HEP‐Test‐Q was utilized consistently showed a significantly diminished physical performance profile in PwH [11, 12].

To date, there is little literature that has investigated distinct determinants that negatively impact the subjective physical performance (SPP) in PwH. Factors that have been associated with a worsened overall SPP include chronic pain persisting for more than 6 months, an impaired joint status, low activity levels, an age over 40 years, and the presence of viral infections (i.e., HAV, HBV) [11, 13]. However, these factors and their impact on SPP have only been analysed in univariate models and with relatively small samples. The latter aspect meant that no analyses could be carried out regarding SPP discriminated for type (A or B) and severity (severe/non‐severe) due to too one‐sided distributions [11]. Therefore, within this study, data for SPP was collected in a large sample of PwH to enable a differentiation between subgroups for disease‐specific parameters (e.g., type, severity), and a more complex statistical model was employed to explore the predictive potential of disease‐specific, person‐related, and arthropathy‐related determinants. This is because, in order to adequately counteract the previously established decline in SPP, the most impactful determinants need to be uncovered.

In detail, the purpose of this study was (1) to investigate the SPP of PwH in comparison to the healthy population, (2) to differentiate SPP on the basis of disease‐specific (e.g., type and severity of haemophilia), person‐related (e.g., age and body mass index), and arthropathy‐related parameters (e.g., pain and joint status) and (3) to identify overarching predictors that negatively affect SPP.

## 2 Materials and Methods

### 2.1 Subjects

A total of 301 patients with mild, moderate or severe haemophilia A or B and 263 healthy controls were included in this multicentre study. The examination period extended from the beginning of 2019 to mid‐2022, and examinations were conducted at the University of Bonn, the Haemophilia Centre in Muenster, and the Department of Sports Medicine in Wuppertal. Eligibility for study participation for both cohorts required an age of at least 18 years, a diagnosis of congenital haemophilia A or B (PwH), the absence of any bleeding disorder (controls), and proficiency in the German language in order to clearly understand the study documents and patient‐reported questionnaires. Subjects were excluded when suffering from (other) bleeding diseases or disorders affecting the joints (e.g., von Willebrand disease, Psoriasis, M. Bechterew). In addition, PwH were excluded if a bleeding event had occurred 2 weeks prior to the examination. PwH were asked to complete questionnaires to collect person‐related and disease‐specific information, as well as data on patients’ SPP and pain. Disease‐specific parameters included, among others, information on the type and severity of haemophilia, treatment characteristics, and concomitant viral diseases. For all centres, approval of the local ethics committees was obtained, and all participants provided written informed consent to participate.

### 2.2 SPP

SPP was assessed by using the widely recognized and validated HEP‐Test‐Q [11, 14]. The HEP‐Test‐Q is a self‐report tool that allows patients to assess self‐perceived levels of physical functioning over the past 4 weeks. The response categories of the items are constructed on a five‐point Likert scale according to frequency, ranging from 1 (never) to 5 (always). The HEP‐Test‐Q consists of four dimensions (mobility, strength and coordination, endurance, and body perception) and a total score, calculated by adding the non‐normalized sums of the sub scores. The sub scores are subsequently normalized, ranging from 0 to 100, with higher values indicating good physical functioning.

### 2.3 Subjective Pain and Clinical Joint Status

Subjectively perceived pain intensity was assessed using the standardized and validated numeric pain rating scale [15], which is part of the German Pain Questionnaire and was distributed to each participant [16]. The 11‐point numerical rating scale ranges from 0 ('no pain') to 10 ('worst pain imaginable'), on which respondents select a whole number that best reflects the intensity of their pain. Pain intensity was assessed for two time points that differentiated between current pain (NRS‐now) and average intensity of pain over the past 4 weeks (NRS‐4w).

Clinical joint status was examined utilizing the Haemophilia Joint Health Score v2.1 (HJHS). This assessment tool was recently revised and validated for adult patients [17]. The HJHS evaluates a patient's joint health on the basis of numerous arthropathy‐related parameters (e.g., swelling, range of motion, muscle atrophy, crepitus). The examination procedure includes the bilateral assessment of the ankle, knee, and elbow joints. Each individual joint has a maximum score of 20 points. In addition, the patient's gait pattern is assessed on a 4‐point scale, leading to a maximum total score of 124 points. Higher scores indicate a worse joint status. Joint assessment was performed by three different investigators of the same research group due to logistical reasons. However, recent research has repeatedly demonstrated a high inter‐rater reliability for the HJHS [18, 19].

### 2.4 Statistics

For a differentiated analysis of the effects of different parameters on the dimensions of SPP and to ensure a coherent structure throughout this paper, the following clusters were formed: disease‐specific (type, severity, treatment, HIV, hepatitis), person‐related (age, body mass index [BMI]), and arthropathy‐related factors (HJHS, NRW‐now, NRS‐4w).

Data was checked for normal distribution by employing the Kolmogorov‐Smirnov test and via visual inspection of Q‐Q plots. Due to the normal distribution of data, Student's t‐tests were used for group comparisons between all PwH and healthy controls as well as for group comparisons between PwH subgroups. Univariate analysis of variance (ANOVA) models were computed to analyse differences in SPP between PwH subgroups and healthy controls. Subgroups for the patient group were formed based on disease‐specific parameters. Effect sizes are presented as partial eta‐squared (*η* ^2^_partial_) for ANOVA (*η* ^2^_partial_ ≥ 0.14 large effect, ≥ 0.06 medium effect, ≥ 0.01 small effect) [20].

Pearson correlations were used to analyse the relationship between SPP dimensions and person‐ and arthropathy‐related variables in PwH. Correlation coefficients were defined as weak, moderate, or strong for coefficient values of *r* = 0.10–0.29, *r* = 0.30–0.49 and *r* = ≥ 0.50, respectively [20].

Finally, to estimate the predictive effect of each individual variable on total SPP in PwH, simple linear regression models were computed. Consequently, each parameter demonstrating a significant impact on SPP was included within a multiple linear regression model to identify the most impactful predictors for the variance of SPP. For this purpose, disease‐specific outcomes were dichotomized in the following manner, with the former category serving as the reference group: type (A, B), severity (non‐severe, severe), treatment (prophylaxis, on demand), HIV (no, yes), and hepatitis (no, yes). Due to lack of homoscedasticity, the heteroscedasticity‐consistent estimator HC3 was employed to ensure no compromise of inference [21]. Statistical analyses were conducted via IBM SPSS 27 (Armonk, NY, USA) for Windows with a predefined alpha level of *p* ≤ 0.05.

## 3 Results

### 3.1 Subjects

The data of 301 PwH and 263 healthy controls were collected and subsequently analysed. A significant difference between PwH and controls was observed for height, joint health, NRS‐4w, and NRS‐now. All characteristics of subjects in both study groups are presented in Table 1.

**TABLE 1:** Demographic and anthropometric data, joint health and pain in patients with haemophilia (PwH) and healthy controls.

Parameter	PwH (*n* = 301) Mean ± SD (min‐max)	Controls (*n* = 263) Mean ± SD (min‐max)	*p* value
Age (years)	42.4 ± 15.1 (18.0–79.0)	41.9 ± 15.1 (19.0–80.0)	0.685
Weight (kg)	84.1 ± 14.4 (53.0–140.0)	83.7 ± 10.7 (57.0–123.0)	0.712
Height (m)	1.80 ± 0.07 (1.57–2.00)	1.82 ± 0.07 (1.67–2.05)	0.005
BMI (kg/m^2^)	25.8 ± 4.2 (18.0–42.3)	25.3 ± 3.1 (19.1–39.7)	0.063
HJHS v2.1^a^	19.2 ± 14.5 (0.0–75.0)	4.7 ± 4.2 (0.0–31.0)	< 0.001
NRS‐now^b^	2.2 ± 2.4 (0.0–10.0)	0.6 ± 1.2 (0.0–7.0)	< 0.001
NRS‐4w^b^	3.1 ± 2.4 (0.0–10.0)	1.0 ± 1.6 (0.0–7.0)	< 0.001

*Note*: Differences are considered significant for *p* ≤ 0.05, employing Student's *t*‐test.

Abbreviations: BMI, body‐mass‐index; HJHS, Haemophilia Joint Health Score version 2.1; NRS‐now, current pain intensity on a numeric rating scale; NRS‐4w, average pain intensity over the last 4 weeks on a numeric rating scale.

^a^ Data for the HJHS were missing from two PwH.

^b^ Data for both pain intensity scales were missing from one PwH.

Among PwH, 86% had haemophilia A, of whom 68% were severely, 17% were moderately and 15% were mildly affected. Of the 14% of PwH B, 61% were severely, 30% moderately and 9% mildly affected. The majority of PwH received prophylaxis, and the subjectively most affected joints were ankles. Clinical data in PwH are presented in Table 2.

**TABLE 2:** Clinical data in patients with haemophilia (*n* = 301).

Parameter	Specification	*n* (%)
Type of haemophilia	A B	258 (86) 43 (14)
Severity	Severe Moderate Mild	201 (67) 56 (19) 44 (15)
Treatment regimen^a^	Prophylaxis On demand	208 (70) 89 (30)
Most affected joints^b^	None Ankle Knee Elbow Others	43 (15) 108 (36) 91 (30) 31 (11) 23 (8)
HIV	No Yes	258 (86) 43 (14)
Hepatitis	No HAV HBV HCV HAV and HCV HBV and HCV HAV and HBV and HCV	243 (81) 3 (1) 7 (2) 30 (10) 1 (1) 6 (2) 11 (3)

Abbreviations: HAV, hepatitis A virus; HBV, hepatitis B virus; HCV, hepatitis C virus.

^a^ Data on treatment regimen was missing from four PwH.

^b^ Data for the subjectively most affected joint was missing from five PwH.

### 3.2 SPP (HEP‐Test‐Q)

**FIGURE 1:**
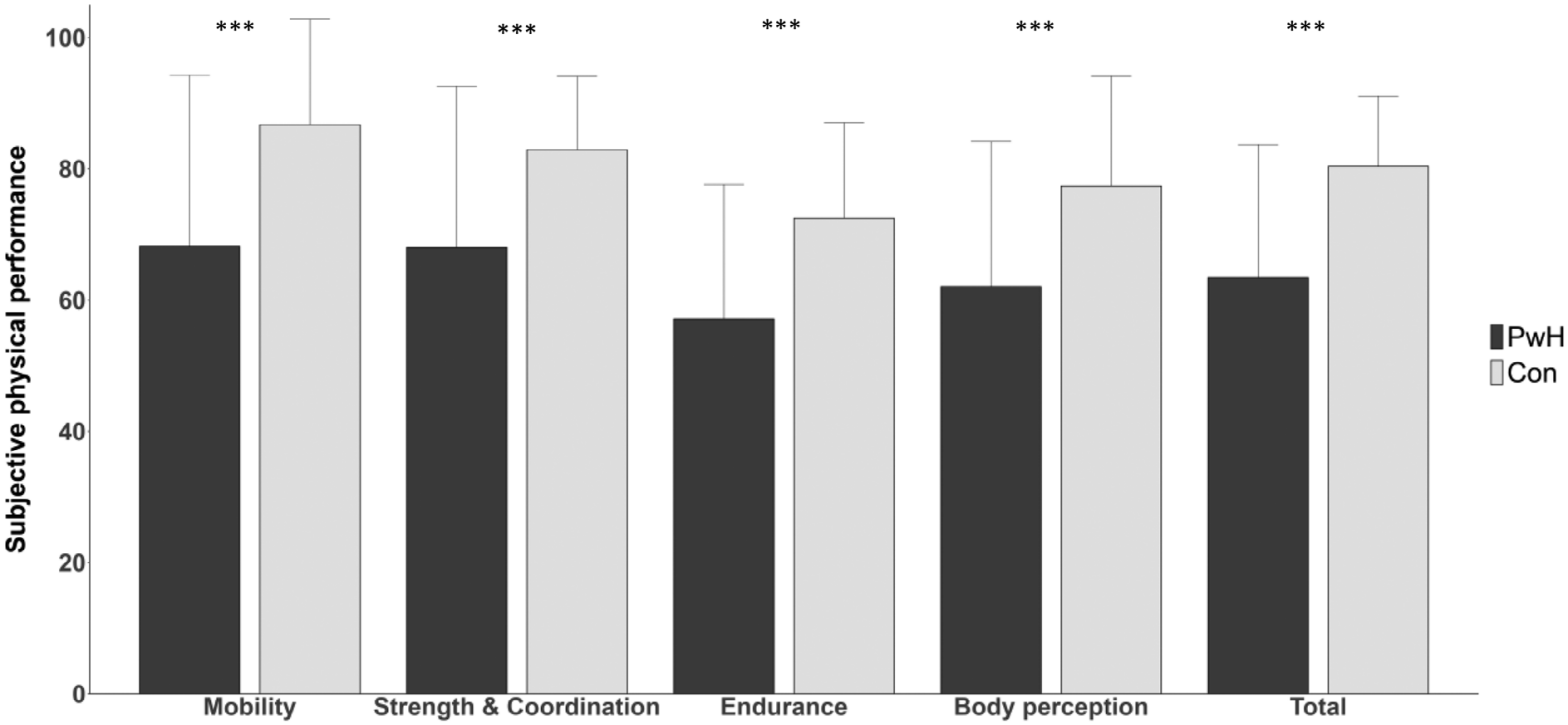
Subjective physical performance (HEP‐Test‐Q) and its dimensions in patients with haemophilia (*n* = 301) and healthy controls (*n* = 263). Data presented as mean and standard deviation (whisker). ^***^ *p* ≤ 0.001, employing Student's *t*‐test.

Results indicate that PwH in general demonstrate a statistically significant deleterious SPP compared to the healthy cohort in terms of the total score and each individual dimension (Figure 1).

### 3.3 Associations Between SPP and Disease‐Specific, Person‐Related, and Arthropathy‐Related Parameters

**FIGURE 2:**
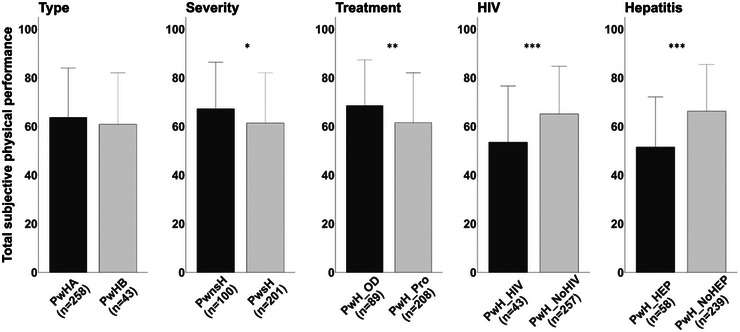
Total score of subjective physical performance (HEP‐Test‐Q) in subgroups of patients with haemophilia (*n* = 301). Data presented as mean and standard deviation (whisker). PwHA, patients with haemophilia A; PwHB, patients with haemophilia B; PwsH, patients with severe haemophilia; PwnsH, patients with non‐severe haemophilia; PwH_Pro, patients with haemophilia on prophylaxis; PwH_OD, patients with haemophilia on demand; PwH_HIV, patients with haemophilia with HIV; PwH_NoHIV, patients with haemophilia without HIV; PwH_HEP, patients with haemophilia with Hepatitis; PwH_NoHep, patients with haemophilia without Hepatitis. ^*^ *p* ≤ 0.05, ^**^ *p* ≤ 0.01, ^***^ *p* ≤ 0.001, employing Student's *t*‐test.

Statistical analyses of between‐group differences for disease‐specific parameters revealed significantly lower values for the total score of SPP and each of the dimensions in subgroups of PwH, compared to controls (*p* < 0.05). Further, between subgroups of PwH no significant differences were observed only for type of haemophilia (*p* = 0.894). Significant subgroup differences were found for severity, treatment, HIV and hepatitis. Subgroup comparisons for the total score of SPP between subgroups of PwH are presented in Figure 2. Additionally, subgroup‐analyses for the different dimensions among subgroups of PwH are presented in Tables S1–S5.

An overview of the correlation analyses is presented in Table 3. In short, person‐related and arthropathy‐related variables all demonstrated significant inverse correlations with each individual dimension of SPP and the total score. For BMI, uniformly weak correlations were observed, while age showed moderate correlations with each dimension of SPP, except for body perception, where a weak correlation was determined. For arthropathy‐related parameters, uniformly moderate correlations were found with endurance and body perception, while moderate to strong correlations were observed with the dimensions of mobility, strength and coordination, and the total score.

**TABLE 3:** Pearson correlation analyses between dimensions and total score of subjective physical performance (HEP‐Test‐Q) and age, BMI, pain and clinical joint status in patients with haemophilia.

Parameter	*n*	Mobility *r* *p* value	Strength and coordination *r* *p* value	Endurance *r* *p* value	Body perception *r* *p* value	Total score *r* *p* value
*Personal*						
Age	301	−0.325 <0.001	−0.472 <0.001	−‐0.335 <0.001	−0.281 <0.001	−0.421 <0.001
BMI	301	−0.156 <0.001	−0.191 <0.001	−0.215 <0.001	−0.200 <0.001	−0.219 <0.001
*Arthropathy*						
NRS‐now	300	−0.506 <0.001	−0.491 <0.001	−0.380 <0.001	−0.354 <0.001	−0.493 <0.001
NRS‐4w	300	−0.615 <0.001	−0.550 <0.001	−0.439 <0.001	−0.428 <0.001	−0.574 <0.001
HJHS	299	−0.505 <0.001	−0.646 <0.001	−0.438 <0.001	−0.309 <0.001	−0.563 <0.001

Abbreviations: BMI, body‐mass‐index; HJHS, Haemophilia Joint Health Score v2.1; NRS‐4w, average pain intensity over the last 4 weeks on a numeric rating scale; NRS‐now, current pain intensity on a numeric rating scale; *r*, Pearson's correlation coefficient.

### 3.4 Results of Univariate and Multiple Regression Analyses

Simple linear regression analyses revealed significant associations for all parameters assessed with the total score of SPP, except for type of haemophilia (*p* = 0.416) (Table 4).

**TABLE 4:** Simple linear regression analyses for each individual predictor in patients with haemophilia.

Predictor	Reference group	*B*	Robust SE	*t*	*p*	95% CI	
						LB	UB
*Personal*							
Age	—	−0.568	0.068	−8.346	<0.001	−0.720	−0.434
BMI	—	−1.060	0.278	−3.817	<0.001	−1.606	−0.513
*Disease*							
Type	A	−2.845	3.496	−0.814	0.416	−9.725	4.035
Severity	NS	−5.817	2.419	−2.405	0.017	−10.578	−1.057
Treatment	PX	6.993	2.459	2.844	0.005	2.154	11.832
HIV	No	−11.617	3.521	−3.299	0.001	−18.547	−4.687
Hepatitis	No	−14.746	3.001	−4.913	<0.001	−20.653	−8.839
*Arthropathy*							
HJHS	—	−0.788	0.058	−13.485	<0.001	−0.903	−0.673
NRS‐now	—	−4.199	0.544	−7.719	<0.001	−5.270	−3.129
NRS‐4w	—	−4.830	0.435	−11.099	<0.001	−5.686	−3.973

*Note*: Dependent variable: subjective physical performance (total score HEP‐Test‐Q) (*n* = 290).

Abbreviations: BMI, body‐mass‐index; HJHS, Haemophilia Joint Health Score v2.1; NRS‐4w, average pain intensity over the last 4 weeks on a numeric rating scale; NRS‐now, current pain intensity on a numeric rating scale; NS, non‐severe; PX, prophylaxis.

Consequently, the type of haemophilia was not included in the multiple linear regression model. The multiple linear regression analysis demonstrated a statistically significant predictive effect of HJHS (*B* = −0.436 [95%‐CI: −0.584; −0.287], *p* < 0.001) and NRS‐4w (*B* = −2.785 [95%‐CI: −4.010; −1.560], *p* < 0.001) on the total score of SPP. The remaining predictors failed to elicit a statistically significant effect (*p* > 0.05). The entire model explained 49% of the variance of the dependent variable (Table 5). Additionally, descriptive data of SPP in relation to the estimated predictive values for the metric predictors, including the estimated regression equations, are available in Table S6.

**TABLE 5:** Multiple linear regression analysis in patients with haemophilia.

Predictor	Reference group	*B*	Robust SE	*β*	*t*	*p*	95% CI	
							LB	UB
Constant	—	94.243	7.181		13.125	<0.001	80.108	108.378
*Personal*								
Age	—	−0.116	0.080	−0.086	−1.452	0.148	−0.274	0.041
BMI	—	−0.365	0.231	−0.077	−1.583	0.115	−0.820	0.089
*Disease*								
Severity	NS	−3.122	3.338	−0.073	−0.935	0.350	−9.692	3.448
Treatment	PX	4.727	3.320	0.107	1.424	0.156	−1.809	11.264
HIV	No	−1.634	3.132	−0.028	−0.522	0.602	−7.799	4.531
Hepatitis	No	−3.269	2.645	−0.064	−1.236	0.217	−8.476	1.937
*Arthropathy*								
HJHS	—	−0.436	0.075	−0.316	−5.773	<0.001	−0.584	−0.287
NRS‐now	—	−0.937	0.690	−0.110	−1.357	0.176	−2.295	0.422
NRS‐4w	—	−2.785	−0.622	−0.329	−4.476	<0.001	−4.010	−1.560

*Note*: Dependent variable: subjective physical performance (total score HEP‐Test‐Q) (*n* = 290). *R* ^2^ = 0.505; adjusted *R* ^2^ = 0.489; *F* (9289) = 31.715, *p* < 0.001.

Abbreviations: BMI, body‐mass‐index; HJHS, Haemophilia Joint Health Score v2.1; NRS‐4w, average pain intensity over the last 4 weeks on a numeric rating scale; NRS‐now, current pain intensity on a numeric rating scale; NS, non‐severe; PX, prophylaxis.

## 4 Discussion

The aim of this study was (1) to explore SPP in PwH compared to the healthy population using the HEP‐Test‐Q, (2) to evaluate the individual impact of disease‐specific, person‐related, and arthropathy‐related parameters on SPP and (3) to determine the most impactful predictors on SPP in univariate and multiple linear regression models.

Firstly, the results indicate a substantial loss in SPP across all dimensions in PwH compared to healthy controls. Previous research has uniformly demonstrated a decline in objectively and subjectively assessed physical performance in PwH [6, 7, 8, 9, 22]. These findings have been so far mainly attributed to maladaptive changes within the musculoskeletal system due to recurrent haemarthroses and decreased participation in physical activity [23, 24]. Additionally, it should be considered that many patients, although no data on the switch to prophylactic treatment were available, were receiving tertiary prophylaxis at the time of data collection and had received on‐demand treatment during the early stages of their life, which likely contributed to the development of haemophilic arthropathy.

Secondly, scores for total SPP significantly differ between subgroups of PwH based on disease severity, treatment regime, and the presence of viral infections (i.e., HIV and hepatitis). Expectedly, no significant differences were observed between PwH A and B. Due to the large sample size that was analysed, statistically reliable group differences for disease‐specific data could be determined for the first time. The findings imply a more deleterious self‐perceived physical functioning in PwH compared to healthy controls, irrespective of the disease severity. Severely affected PwH are genetically more predisposed to spontaneous haemorrhage and bleeding events in general. Unfortunately, despite remarkable improvements in replacement therapy in the past decades, bleeding, regardless of severity, can currently not be fully prevented [25]. Hence, the manifestation of arthropathy might still occur, especially since recent data increasingly support the occurrence of subclinical bleedings, which presumably occur more frequently in severely affected patients [3]. The substantial difference in SPP between severity types might also be explained by the overall level of physical activity and sports participation, although this was not directly assessed in the present investigation. However, a recently published systematic review concluded, although with not entirely consistent results, that patients with severe haemophilia participate less frequently in physical activity while generally choosing lower intensity levels [26]. Data analyses also revealed a more pronounced decline in SPP for patients on prophylaxis compared to patients receiving on‐demand treatment. This association is likely mediated by the rational tendency to disproportionately administer prophylactic treatment to patients with the severe genotype or with a higher haemorrhage incidence. Accordingly, this correlation, which at first glance appears somewhat counterintuitive, can be explained rather indirectly by the increased bleeding tendency of severely affected patients than directly by the treatment modality administered. Whether SPP varies by type of haemophilia has not yet been studied. The present results suggest that PwH A and B have statistically comparable values. Considering that no difference was found between HJHS (*p* = 0.516) and NRS‐4w (*p* = 0.712) for type of haemophilia in our study and elsewhere for patients with moderate haemophilia [27], and those predictors most potently determine SPP, comparable results are to be expected. Interestingly, between‐group analyses of the individual dimensions of SPP revealed significant differences among subgroups of PwH for most disease‐specific parameters; however, not regarding *body perception* (see Table S5). This may be explained by the fact that *body perception* represents the only dimension primarily related to self‐confidence and trust in one's own physical abilities rather than the direct perception of physical functioning. This finding, though, requires further investigation. In addition, correlation analyses also demonstrated a reasonable association of age, BMI, pain and clinical joint status with all dimensions of SPP, which are discussed below together with the results of the regression analyses.

Thirdly, simple linear regression models demonstrate a significant association between all disease‐specific, person‐related, and arthropathy‐related parameters, except for type of haemophilia and total SPP. This implies, in theory, that total SPP could indeed be affected by various factors that may be part of a patient's life. When all parameters were transferred into one complex statistical model, only the average perceived pain intensity (NRS‐4w) and HJHS retained their statistical significance, indicating their overriding role in explaining the variance of SPP in PwH. This discrepancy can be explained by the concept of confounding. For instance, patients with viral comorbidities tend to be of older age, as contaminated plasma products were occasionally administered mainly in the 1980s [28], and age represents a factor affecting SPP in and of itself. Therefore, HIV and hepatitis only indirectly affect SPP; in reality, SPP is determined rather by the patient's age. However, older PwH typically exhibit worse joint conditions [29]. Thus, the association between age and SPP is confounded by the patient's joint status, explaining the results of the multiple regression analysis. In this vein, disease‐specific parameters such as type and severity of haemophilia should also be considered. Patients with severe haemophilia are, as mentioned before, more frequently affected by arthropathic alterations [30] and experience pain more often [31], particularly if prophylactic treatment was not initiated early in life. This contributes to the indirect effect on SPP observed in the statistical model, as no significant direct effect was found within the multiple regression analysis. Instead, this indirect effect is rather mediated through arthropathy‐related factors, explaining the absence of a direct association.

Furthermore, unlike the average reported pain intensity (NRS‐4w), acute pain intensity (NRS‐now) showed no independent effect on SPP. A possible explanation for this could be the chronic burden of persistent pain on both physical and psychological health, which may not necessarily be conveyed through acute pain sensation. Given that the outcome (i.e., SPP) represents a subjective measure, it is plausible that prolonged maladaptation related to pain and also arthropathy has a stronger impact on an individual's perceived physical capacity.

It should be noted, however, that an individual's pain perception is inherently multifactorial. Research suggests that some patients, including PwH, exhibit altered pain perception even in the absence of arthropathy [32, 33], while the relationship between pain and functional ability may vary depending on the context [34, 35], further emphasizing the complexity of pain as it relates to physical functioning. This conclusion does not imply that predictors other than HJHS and NRS‐4w have no impact on a patient's SPP, but that the variation in SPP is predominantly attributed to the joint condition and average pain perception in PwH. It's noteworthy that the multiple regression model explained approximately half of the variance in SPP (49%), demonstrating not only a strong statistical model but also highlighting the relevance of the included predictors in accounting for variations in SPP. Nonetheless, additional factors must be considered, as only half of the variance of SPP is explained by the variables included in the presented statistical model. It seems reasonable to consider all dimensions of the biopsychosocial model—despite its primary use in conceptualizing interindividual differences in pain—as putative sources of an impaired SPP. Consequently, one might speculate that psychological factors (e.g., depressive symptoms, pain catastrophizing, kinesiophobia), factors associated with social support (e.g., friendships, partnerships), and behavioural aspects (e.g., sleep quality) may further influence the self‐perceived physical functioning. Given the rather physiological focus of the available data, this article aimed at addressing SPP from a biomedical perspective. Future research should therefore seek to identify the remaining factors affecting SPP that have been alluded to but were not explicitly explored.

Fortunately, joint status and pain are modifiable variables, unlike predictors such as age, viral comorbidities, or severity of the disease. Advances in factor replacement therapy, recent WHO guideline updates encouraging more exercise even for individuals with disabilities [36, 37], and the continued decline in ABR provide optimism that SPP levels might be aligned with the healthy population once arthropathy and persistent pain conditions could be prevented or further minimized. However, while advancements in haemophilia treatment have facilitated greater engagement in physical activity, PwH still do not consistently meet WHO recommendations [26]. This implies that barriers (e.g., fear of injury, accessibility of tailored exercise programs) still limit physical activity participation. Hence, improvements in SPP related to disease‐related parameters, despite being beneficial, do not fully account for limitations in physical activity levels.

To achieve SPP levels comparable to the general population, the early administration of prophylactic therapy to prevent haemophilic arthropathy and associated comorbidities, as well as the establishment of a regular physical activity regimen in PwH, is mandatory. In particular, early and adequate prophylaxis is pivotal to preserve joint integrity and musculoskeletal health [38]. This enables PwH to participate in regular physical activity to promote a healthy lifestyle that reduces the risk of developing metabolic and/or cardiovascular diseases that are associated with low levels of physical activity [39].

As a last aspect worth mentioning, the HEP‐Test‐Q should be used on an ongoing basis in PwH to gain a comprehensive impression of a patient's self‐perceived functional profile. Not only do the distinct dimensions of SPP correlate well with objective measures such as the 12‐min walk test and one‐leg stand [12], but the subjective nature of the questionnaire additionally allows a deeper insight into the patient's self‐efficacy, among others, in terms of functional capacity. Finally, in order to investigate the positive impact of the development of medical care on all domains of psychological and physiological well‐being, the HEP‐Test‐Q should be utilized in future research as one critical mosaic of factors that predicts health‐related quality of life.

### 4.1 Strengths and Limitations

A major strength of this study is the large and thus representative sample size considering the rare nature of the disease. However, some limitations are worth mentioning. We chose to use a numerical pain scale for pain assessment, including chronic pain states, because, on one hand, only one disease‐specific questionnaire currently exists in the field of haemophilia. This questionnaire was developed in Portugal, and its content was not considered suitable for our cohort [40]. On the other hand, we aimed to use a measurement instrument that is widely used in Germany. However, current literature suggests that chronic pain may not be accurately captured by a numerical number [41, 42], and this limitation should be considered when interpreting the results. Data on annual bleeding rates and objective levels of physical activity were not considered in the analyses. This is due to no or only partial availability of data. The impact of annual bleeding rates was vicariously evaluated by linking the orthopaedic joint score as a clinical correlate of recurrent haemarthroses to the SPP. Whether bleeding rates and physical activity are directly correlated to SPP requires further investigation. Furthermore, the extent to which psychological factors such as kinesiophobia or depression impact SPP was not recorded or analysed.

## 5 Conclusion

Despite more elaborate treatment options to address factor deficiency in PwH, the physical performance profile is still majorly impaired. The decreased SPP can be attributed primarily and independently to arthropathy‐related factors such as an impaired joint status and increased pain levels that persist over an extended period of time. As the manifestation and progression of haemophilic arthropathy are induced by articular bleeding, the main focus should be on preventing bleeding events from happening. To oppose the decline in physical proficiency, personalized sports‐therapeutic programs should be part of the multimodal treatment concept and implemented in everyday life [43].

## Author Contributions

A.St. and T.H. designed the study. S.V.M. and T.H. developed the HEP‐Test‐Q questionnaire in a previous study. A.St., F.T., P.R., M.B. performed the data collection and A.St. and F.T. performed the data analysis. ASt., H.R., J.O. supported with the recruitment process and provided scientific expertise together with F.T. and T.H. A.St., F.T., and T.H. drafted the initial manuscript. T.H. additionally supervised the project. All authors critically revised and approved the final version of the manuscript.

## Ethics Statement

This study was approved by the local Ethical Review Boards (University of Wuppertal MS/BB 180613; Westphalia Lippe Medical Association: 2018‐510‐f‐S; University Hospital Bonn: 339/19).

## Conflicts of Interest

Sylvia von Mackensen has no known competing interests to declare. Alexander Schmidt has received speakers’ fees from Sobi and Takeda. Fabian Tomschi has received speakers’ fees from Takeda and received an educational grant from Sobi. Pia Ransmann has received speakers’ fees and travel support from Takeda as well as travel support from Sobi. Marius Brühl has received travel fees from Sobi and Takeda. Andreas C. Strauss has received research funding from Bayer, Sobi and Takeda and has received consultancy, speakers’ bureau, honoraria, scientific advisory board and travel expenses from Bayer, Biotest, CSL Behring, Novo Nordisk, Sobi and Takeda. Heinrich Richter has received research and travel grants from Bayer, Biotest, CSL Behring, Intersero, Novo Nordisk, Pfizer, Sobi and Takeda. Johannes Oldenburg has received research funding from Bayer, Biotest, CSL Behring, Octopharma, Pfizer, Sobi and Takeda as well as honoraria (for consultancy, speakers’ bureau, scientific advisory board) and/or travel expenses from Bayer, Biogen Idec, Biomarin, Biotest, Chugai, CSL Behring, Freeline, Grifols, LFB, Novo Nordisk, Octopharma, Pfizer, Roche, Sanofi, Spark Therapeutics, Sobi, and Takeda. Thomas Hilberg has received research funding from Biotest, Chugai, CSL Behring, Intersero, Roche, Sobi and Takeda as well as travel expenses, speakers’ or scientific advisory board honoraria from Bayer, Biotest, Chugai, Novo Nordisk, Pfizer, Roche, Sanofi, Sobi and Takeda.

## Patient Consent

Written informed consent from each participant was obtained by the respective investigative site before enrolment in the study.

## Supporting information



Supporting Information

## Data Availability

The data that support the findings of this study are available from the corresponding author upon reasonable request.
