# Supplementary material for: Subjective Physical Performance and Its Determinants in Patients With Haemophilia
Source: Haemophilia. 2025 Mar 28;31(3):535–43. doi: 10.1111/hae.70037 (PMC12175108; doi:10.1111/hae.70037)
Supplement: Supplementary file 1 — Supporting Information [file HAE-31-535-s001.docx]

**Supplementary Table 1.** Impact of disease-related outcomes on total subjective physical performance in subgroups of patients with haemophilia and healthy controls (Con)

| **Para-**  **meter** | **Groups** | **n** | **Mean ± SD (min-max)** | **ANOVA**  **p-value** | **Effect size** | **Group**  **comparison** | **Post-hoc**  **p-value** |
| --- | --- | --- | --- | --- | --- | --- | --- |
| Type | A  B  Con | 258  43  263 | 63.8 ± 20.2 (20.0-100.0)  60.9 ± 21.1 (21.0-96.0)  80.4 ± 10.7 (42.0-99.0) | <.001 | .210 | A vs B  A vs Con  B vs Con | .894  <.001  <.001 |
| Severity | S  NS  Con | 201  102263 | 61.4 ± 20.7 (20.0-100.0)  67.2 ± 19.2 (21.0-100.0)  80.4 ± 10.7 (42.0-99.0) | <.001 | .220 | S vs NS  S vs Con  NS vs Con | .012  <.001  <.001 |
| Treatment | PRO  OD  Con | 208  89  263 | 61.6 ± 20.5 (20.0-100.0)  68.6 ± 18.8 (21.0-100.0)  80.4 ± 10.7 (42.0-99.0) | <.001 | .220 | PRO vs OD  PRO vs Con  OD vs Con | .002  <.001  <.001 |
| HIV | No  Yes  Con | 257  43  263 | 65.2 ± 19.6 (20.0-100.0)  53.5 ± 21.4 (20.0-96.0)  80.4 ± 10.7 (42.0-99.0) | <.001 | .233 | No vs Yes  No vs Con  Yes vs Con | <.001  <.001  <.001 |
| Hepatitis | No  Yes  Con | 239  58  263 | 66.3 ± 19.2 (21.0-100.0)  51.5 ± 20.6 (20.0-96.0)  80.4 ± 10.7 (42.0-99.0) | <.001 | .261 | No vs Yes  No vs Con  Yes vs Con | <.001  <.001  <.001 |

Data presented as mean ± standard deviation (Min-Max).
A = patients with haemophilia A, B = patients with haemophilia B, S = patients with severe haemophilia, NS = patients with non-severe haemophilia, PRO = patients on prophylaxis treatment, OD = patients on on-demand treatment.
Differences are considered significant for p ≤ .05, employing ANOVA with Bonferroni-adjustment.

**Supplementary Table 2.** Impact of disease-related outcomes on mobility in subgroups of patients with haemophilia and healthy controls (Con)

| **Para-**  **meter** | **Groups** | **n** | **Mean ± SD (min-max)** | **ANOVA**  **p-value** | **Effect size** | **Group**  **comparison** | **Post-hoc**  **p-value** |
| --- | --- | --- | --- | --- | --- | --- | --- |
| Type | A  B  Con | 258  43  263 | 68.6 ± 25.8 (6.3-100.0)  65.8 ± 28.2 (12.5-100.0)  86.7 ± 16.1 (31.3-100.0) | <.001 | .151 | A vs B  A vs Con  B vs Con | .999  <.001  <.001 |
| Severity | S  NS  Con | 201  102263 | 65.8 ± 26.2 (6.3-100.0)  73.0 ± 25.6 (12.5-100.0)  86.7 ± 16.1 (31.3-100.0) | <.001 | .161 | S vs NS  S vs Con  NS vs Con | .022  <.001  <.001 |
| Treatment | PRO  OD  Con | 208  89  263 | 66.6 ± 26.2 (6.3-100.0)  73.0 ± 25.5 (18.8-100.0)  86.7 ± 16.1 (31.3-100.0) | <.001 | .154 | PRO vs OD  PRO vs Con  OD vs Con | .062  <.001  <.001 |
| HIV | No  Yes  Con | 257  43  263 | 70.1 ± 26.1 (6.3-100.0)  58.1 ± 23.1 (18.8-100.0)  86.7 ± 16.1 (31.3-100.0) | <.001 | .165 | No vs Yes  No vs Con  Yes vs Con | .003  <.001  <.001 |
| Hepatitis | No  Yes  Con | 239  58  263 | 74.5 ± 25.1 (12.5-100.0)  54.8 ± 25.7 (6.3-100.0)  86.7 ± 16.1 (31.3-100.0) | <.001 | .192 | No vs Yes  No vs Con  Yes vs Con | <.001  <.001  <.001 |

Data presented as mean ± standard deviation (Min-Max).
A = patients with haemophilia A, B = patients with haemophilia B, S = patients with severe haemophilia, NS = patients with non-severe haemophilia, PRO = patients on prophylaxis treatment, OD = patients on on-demand treatment.
Differences are considered significant for p ≤ .05, employing ANOVA with Bonferroni-adjustment.

**Supplementary Table 3.** Impact of disease-related outcomes on strength & coordination in subgroups of patients with haemophilia and healthy controls (Con)

| **Para-**  **meter** | **Groups** | **n** | **Mean ± SD (min-max)** | **ANOVA**  **p-value** | **Effect size** | **Group**  **comparison** | **Post-hoc**  **p-value** |
| --- | --- | --- | --- | --- | --- | --- | --- |
| Type | A  B  Con | 258  43  263 | 68.4 ± 24.3 (9.4-100.0)  66.0 ± 25.8 (12.5-100.0)  82.9 ± 11.2 (21.9-100.0) | <.001 | .128 | A vs B  A vs Con  B vs Con | .999  <.001  <.001 |
| Severity | S  NS  Con | 201  102263 | 64.9 ± 25.3 (12.5-100.0)  74.3 ± 21.7 (9.4-100.0)  82.9 ± 11.2 (21.9-100.0) | <.001 | .152 | S vs NS  S vs Con  NS vs Con | <.001  <.001  <.001 |
| Treatment | PRO  OD  Con | 208  89  263 | 65.5 ± 24.8 (21.5-100.0)  75.3 ± 21.9 (9.4-100.0)  82.9 ± 11.2 (21.9-100.0) | <.001 | .148 | PRO vs OD  PRO vs Con  OD vs Con | <.001  <.001  .004 |
| HIV | No  Yes  Con | 257  43  263 | 70.5 ± 23.6 (9.4-100.0)  54.1 ± 25.2 (12.5-96.9)  82.9 ± 11.2 (21.9-100.0) | <.001 | .166 | No vs Yes  No vs Con  Yes vs Con | <.001  <.001  <.001 |
| Hepatitis | No  Yes  Con | 239  58  263 | 72.1 ± 23.0 (9.4-100.0)  51.7 ± 24.2 (12.5-96.9)  82.9 ± 11.2 (21.9-100.0) | <.001 | .207 | No vs Yes  No vs Con  Yes vs Con | <.001  <.001  <.001 |

Data presented as mean ± standard deviation (Min-Max).
A = patients with haemophilia A, B = patients with haemophilia B, S = patients with severe haemophilia, NS = patients with non-severe haemophilia, PRO = patients on prophylaxis treatment, OD = patients on on-demand treatment.
Differences are considered significant for p ≤ .05, employing ANOVA with Bonferroni-adjustment.

**Supplementary Table 4.** Impact of disease-related outcomes on endurance in subgroups of patients with haemophilia and healthy controls (Con)

| **Para-**  **meter** | **Groups** | **n** | **Mean ± SD (min-max)** | **ANOVA**  **p-value** | **Effect size** | **Group**  **comparison** | **Post-hoc**  **p-value** |
| --- | --- | --- | --- | --- | --- | --- | --- |
| Type | A  B  Con | 258  43  263 | 57.7 ± 20.3 (9.4-100.0)  53.9 ± 22.0 (6.3-96.9)  72.5 ± 14.6 (25.0-100.0) | <.001 | .156 | A vs B  A vs Con  B vs Con | .597  <.001  <.001 |
| Severity | S  NS  Con | 201  102263 | 55.8 ± 21.1 (6.3-100.0)  59.8 ± 19.2 (18.8-100.0)  72.5 ± 14.6 (25.0-100.0) | <.001 | .159 | S vs NS  S vs Con  NS vs Con | .213  <.001  <.001 |
| Treatment | PRO  OD  Con | 208  89  263 | 55.5 ± 20.8 (6.3-100.0)  62.2 ± 18.7 (18.8-100.0)  72.5 ± 14.6 (25.0-100.0) | <.001 | .163 | PRO vs OD  PRO vs Con  OD vs Con | .008  <.001  <.001 |
| HIV | No  Yes  Con | 257  43  263 | 58.6 ± 19.7 (12.5-100.0)  49.3 ± 23.3 (6.3-93.8)  72.5 ± 14.6 (25.0-100.0) | <.001 | .167 | No vs Yes  No vs Con  Yes vs Con | .005  <.001  <.001 |
| Hepatitis | No  Yes  Con | 239  58  263 | 59.3 ± 19.4 (18.8-100.0)  48.0 ± 22.6 (6.3-93.8)  72.5 ± 14.6 (25.0-100.0) | <.001 | .182 | No vs Yes  No vs Con  Yes vs Con | <.001  <.001  <.001 |

Data presented as mean ± standard deviation (Min-Max).
A = patients with haemophilia A, B = patients with haemophilia B, S = patients with severe haemophilia, NS = patients with non-severe haemophilia, PRO = patients on prophylaxis treatment, OD = patients on on-demand treatment.
Differences are considered significant for p ≤ .05, employing ANOVA with Bonferroni-adjustment.

**Supplementary Table 5.** Impact of disease-related outcomes on body perception in subgroups of patients with haemophilia and healthy controls (Con)

| **Para-**  **meter** | **Groups** | **n** | **Mean ± SD (min-max)** | **ANOVA**  **p-value** | **Effect size** | **Group**  **comparison** | **Post-hoc**  **p-value** |
| --- | --- | --- | --- | --- | --- | --- | --- |
| Type | A  B  Con | 258  43  263 | 62.3 ± 22.1 (10.0-100.0)  60.1 ± 22.6 (10.0-100.0)  77.4 ± 16.7 (10.0-100.0) | <.001 | .133 | A vs B  A vs Con  B vs Con | .999  <.001  <.001 |
| Severity | S  NS  Con | 201  102263 | 61.4 ± 22.3 (10.0-100.0)  63.2 ± 22.0 (10.0-100.0)  77.4 ± 16.7 (10.0-100.0) | <.001 | .133 | S vs NS  S vs Con  NS vs Con | .999  <.001  <.001 |
| Treatment | PRO  OD  Con | 208  89  263 | 61.2 ± 22.7 (10.0-100.0)  64.5 ± 20.7 (20.0-100.0)  77.4 ± 16.7 (10.0-100.0) | <.001 | .132 | PRO vs OD  PRO vs Con  OD vs Con | .565  <.001  <.001 |
| HIV | No  Yes  Con | 257  43  263 | 63.2 ± 21.3 (15.0-100.0)  55.6 ± 25.2 (10.0-100.0)  77.4 ± 16.7 (10.0-100.0) | <.001 | .140 | No vs Yes  No vs Con  Yes vs Con | .055  <.001  <.001 |
| Hepatitis | No  Yes  Con | 239  58  263 | 64.0 ± 21.6 (10.0-100.0)  54.3 ± 22.4 (20.0-100.0)  77.4 ± 16.7 (10.0-100.0) | <.001 | .149 | No vs Yes  No vs Con  Yes vs Con | .002  <.001  <.001 |

Data presented as mean ± standard deviation (Min-Max).
A = patients with haemophilia A, B = patients with haemophilia B, S = patients with severe haemophilia, NS = patients with non-severe haemophilia, PRO = patients on prophylaxis treatment, OD = patients on on-demand treatment.
Differences are considered significant for p ≤ .05, employing ANOVA with Bonferroni-adjustment.

**Supplementary Table 6.** Descriptive data of subjective physical performance (SPP) and estimated predictive values for age, BMI, HJHS, NRS-now, NRS-4w based on linear regression analyses in patients with haemophilia (n=301)

| Predictor | Estimated  regression equation | Minimum / predicted SPP | 25%-  Percentile / predicted SPP | 50%-  Percentile / predicted SPP | 75%-  Percentile / predicted SPP | Maximum / predicted SPP |
| --- | --- | --- | --- | --- | --- | --- |
| Age | y=87.40-0.57*x | 18 / 77.14 | 29 / 70.87 | 41 / 64.03 | 55 / 56.05 | 80 / 41.80 |
| BMI | y=90.74-1.06*x | 18.0 / 71.66 | 23.2 / 66.15 | 25.1 / 64.13 | 27.5 / 61.59 | 42.3 / 45.90 |
| HJHS^α^ | y=78.57-0.79*x | 0 / 78.57 | 4 / 75.41 | 9 / 71.46 | 21 / 61.98 | 75 / 19.32 |
| NRS-now^β^ | y=72.42-4.20*x | 0 / 72.42 | 0 / 72.42 | 0 / 72.42 | 2 / 64.02 | 10 / 30.42 |
| NRS-4w^β^ | y=78.16-4.83*x | 0 / 78.16 | 0 / 78.16 | 2 / 68.50 | 4 / 58.84 | 10 / 29.86 |

^α^n = 299, ^β^n = 300.
